# P-2115. Seroprevalence of Strongyloides stercoralis amongst pediatric solid organ transplant candidates at a tertiary pediatric medical center in the United States

**DOI:** 10.1093/ofid/ofaf695.2279

**Published:** 2026-01-11

**Authors:** Victoria Liu, Caitlin N Brammer, William R Otto, Maryam Mysorewala, Mark Murphy, Grant C Paulsen, Lara A Danziger-Isakov

**Affiliations:** University of Cincinnati School of Medicine, Cincinnati, Ohio; Cincinnati Children's Hospital Medical Center, Cincinnati, OH; Cincinnati Children's Hospital Medical Center, Cincinnati, OH; Cincinnati Children's Hospital Medical Center, Cincinnati, OH; Cincinnati Children's Hospital Medical Center, Cincinnati, OH; Cincinnati Children's Hospital Medical Center, Cincinnati, OH; Cincinnati Children's Hospital, Cincinnati, OH

## Abstract

**Background:**

*Strongyloides stercoralis* is an intestinal nematode endemic to Appalachia and the southeastern United States. Solid organ transplant (SOT) recipients are at risk of *Strongyloides* hyperinfection, characterized by massive parasite burden and disseminated infection. Screening of SOT candidates with risk factors for strongyloidiasis is recommended to prevent infection. The prevalence of *Strongyloides* infection in the United States and how to identify at-risk patients is not well understood. This study sought to define Strongyloides seropositivity in pediatric SOT candidates in a region not considered endemic for infection.

**Table 1:**
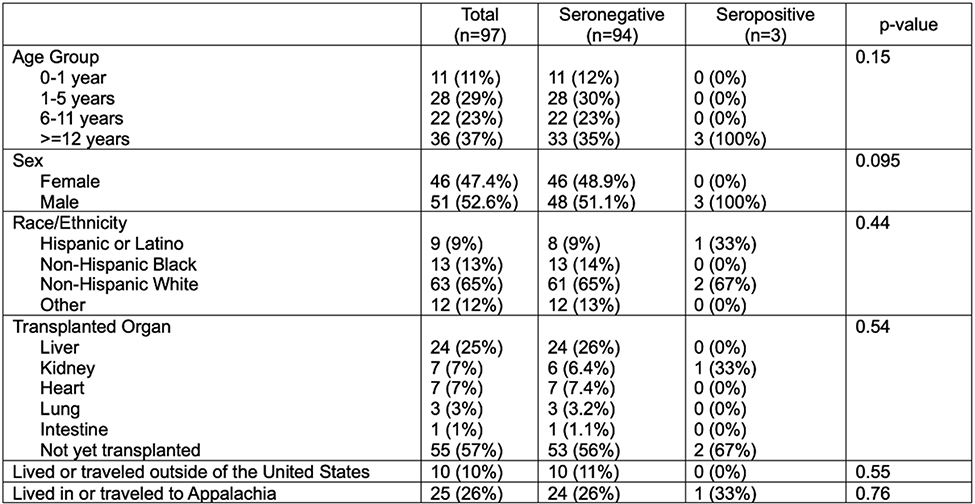
Demographic and clinical characteristics of patients in the cohort

**Methods:**

This was a retrospective study of patients who underwent evaluation for SOT during 2023-2024 at Cincinnati Children’s Hospital Medical Center, where all SOT candidates undergo screening for *Strongyloides* infection. Demographic and clinical data were collected and described. Variables were compared between groups using the Chi-square test and Wilcoxon rank sum test for continuous variables.

**Results:**

A total of 97 patients underwent evaluation for SOT. The cohort was majority White, non-Hispanic males (Table 1). *Strongyloides* seropositivity was noted in 3/97 patients (3.1%, 95% confidence interval 0.6%-8.7%), only one of which had traveled to or lived in Appalachia. When excluding children < 2 years of age, 3/69 (4.3%, 95% CI 0.9-12.2%) were seropositive. Overall, 25/97 (25.8%) of patients in the cohort had exposure to the Appalachian region, while 10/97 (10.3%) were born, lived in, or traveled to other countries. Between seropositive and seronegative patients, there was no difference in exposure to Appalachian regions or other risk factors of *Strongyloides* infection. Seropositive patients were more likely to have eosinophilia (2/3 versus 16/94, p = 0.03). Other clinical signs or symptoms of infection were similar between groups.

**Conclusion:**

In this cohort of pediatric SOT candidates cared for at a transplant center adjacent to an endemic region, *Strongyloides -*seropositivity was low. There were seropositive patients without classic risk factors for Strongyloides infection. Universal screening for *Strongyloides* in SOT candidates > 2 years of age should be considered to minimize risk of *Strongyloides* hyperinfection in at-risk patients.

**Disclosures:**

Grant C. Paulsen, MD, Moderna, Inc: Grant/Research Support|Pfizer: Grant/Research Support|Sanofi: Grant/Research Support Lara A. Danziger-Isakov, MD, MPH, Aicuris: Grant/Research Support|Ansun BioPharma: Grant/Research Support|Astellas: Advisor/Consultant|Astellas: Grant/Research Support|Merck: Advisor/Consultant|Merck: Grant/Research Support|Pfizer (Any division): Grant/Research Support|Takeda: Grant/Research Support

